# Factors associated with change in the need for recovery and subjective listening effort in employees with hearing loss receiving aural rehabilitation

**DOI:** 10.1007/s00420-022-01920-1

**Published:** 2022-09-12

**Authors:** Hanneke E. M. van der Hoek-Snieders, Monique Boymans, Wouter A. Dreschler

**Affiliations:** Amsterdam UMC, Department of ENT-Audiology, University of Amsterdam, Meibergdreef 9, 1105 AZ Amsterdam, The Netherlands

**Keywords:** Need for recovery, Workers, Fatigue, Hearing loss, Listening effort, Aural rehabilitation

## Abstract

**Objective:**

Compared to normally-hearing employees, those with hearing loss suffer from higher Need For Recovery (NFR) after work. The aims of this study are to assess the NFR of employees with hearing loss before and after aural rehabilitation and to examine to what extent change in the NFR can be explained by changes in subjective listening effort, personal adjustments, communication strategies, auditory work demands, and self-reported hearing ability.

**Methods:**

We included patients who received aural rehabilitation in two audiological centers in the Netherlands because of hearing complaints in their work situation. Outcomes were measured by questionnaires at baseline and 3 month follow-up. The NFR before and after the rehabilitation was compared with a *t* test. Hierarchical multiple analyses were performed.

**Results:**

In total, 60 patients (aged 22–63, working hours ≥8 per week) participated in the study, of which 50 completed the follow-up questionnaires. The NFR was significantly lower after the aural rehabilitation (*M* = 45.03) compared to before the aural rehabilitation (*M* = 51.89), *t* = −3.43, *p* < 0.01). Change in NFR could best be explained by the change in personal adjustments (*R*^2^ = 0.45, *B* = −1.23, *p* < 0.01).

**Conclusion:**

The NFR of employees with hearing loss can be improved by aural rehabilitation, but this study shows that current practices reduce the NFR only in part of the employees. Therefore, improving current practices should be considered and evaluated, for example by applying a different combination of rehabilitation components. Especially, interventions that affect personal adjustments may be promising to further reduce the NFR in employees with hearing loss.

## Introduction

Hearing loss is a prevalent health problem and can severely affect the well-being and work functioning of employees (Danermark and Gellerstedt 2004). It causes more effort and concentration to be required to perform auditory job tasks, such as communicating with colleagues or responding to auditory warning signals (Tufts et al. 2009). Sustained listening under difficult conditions—such as noisy workplaces or workplaces with reverberation—can be demanding and fatiguing (Holman et al. 2019; Hornsby et al. 2016). Compared to normally hearing employees, those with hearing loss experience more intense fatigue, and/or require a longer period to recuperate from work-induced fatigue (Holman et al. 2021a; Nachtegaal et al. 2012, 2009). In other words, their Need For Recovery (NFR) after work is generally higher.

NFR is not only an indicator of work-induced fatigue, but also a predictor of stress, subjective health complaints, and sickness leave (de Croon et al. 2003; Sluiter et al. 2003). Assessing the NFR can therefore be used to screen for employees at risk for occupational diseases (Broersen et al. 2004). Employees with hearing loss are more likely to have reduced work productivity, to take more sickness leave, to become unemployed, and to take earlier retirement (Danermark and Gellerstedt 2004; Helvik et al. 2013; Mohr et al. 2000; Nachtegaal et al. 2012; Shan et al. 2020). Monitoring the NFR of employees with hearing loss may therefore be valuable to identify employees at risk for occupational diseases. Monitoring can also be used to evaluate the effects of interventions that aim to reduce hearing complaints in work situations and to improve work participation in individuals with hearing loss.

**Fig. 1:**
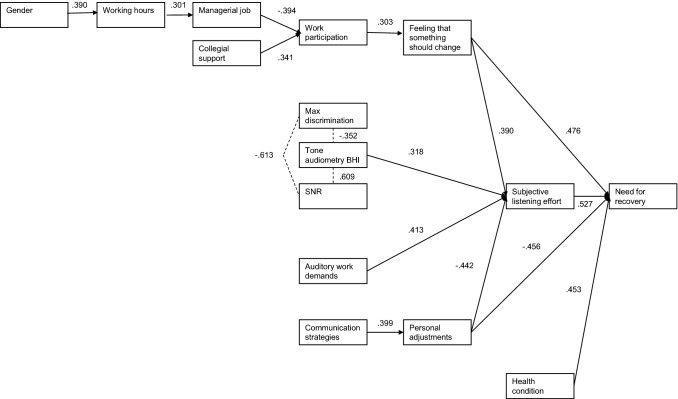
Model of factors influencing the Need For Recovery (NFR) of employees with hearing loss as proposed by van der Hoek-Snieders et al. (2020)

Recently, there has been an increasing interest in the NFR of employees with hearing loss. In our previous study (van der Hoek-Snieders et al. 2020), NFR and the underlying relationships with several hearing-related, work-related, and personal factors were assessed in 294 employees with hearing loss. A model was proposed of factors influencing the NFR in this population (Fig. 1).

NFR is influenced by subjective listening effort (LE) and some factors influence both NFR and LE according to this model. Specifically, NFR and LE are influenced by the feeling that something should change at work and by making personal adjustments, which include self-acceptance, acceptance of loss, and stress and withdrawal. It was found that “feeling that something should change” and a poorer ability to make personal adjustments were associated with higher NFR and higher LE.

Differences between the constructs NFR and LE were also reported in that study. According to the model, NFR is influenced by employees’ general health condition, but LE is not. Reporting a moderate or poor health condition, rather than a good health condition, was found to correlate moderately with a higher NFR. Furthermore, it was found that LE is influenced by employees’ hearing status measured with pure-tone audiometry, but NFR is not. Earlier studies report inconsistent results regarding the association between hearing status and NFR (Nachtegaal et al. 2009; Wang et al. 2018).

It should be noted that the hypothesized model of our previous study (van der Hoek-Snieders et al. 2020) has been constructed based on the correlations found in their study sample, and the model has not yet been validated in an independent sample. Also, this previous study was based on cross-sectional data. Therefore, the data do not allow strong statements about the causality regarding the effect of the interventions on the NFR of employees with hearing loss.

Most interventions provided to employees with hearing loss can be captured within the domain of aural rehabilitation. The aim of aural rehabilitation is to reduce hearing complaints in social life and in work situations and to improve work participation and daily life functioning (Boothroyd 2007, 2017). It can consist of four components, respectively, sensory management (e.g., the provision of hearing aids), perceptual training, instruction, and counselling. Instruction is a more directive manner of psycho-education, whereas counselling is more person-centered.

Recently, Granberg and Gustafsson (2021) concluded in a scoping review that the literature regarding rehabilitation services for employees with hearing loss is scarce. It is for example not well described which disciplines should be involved or which specific services should be provided. In the Netherlands, individually tailored aural rehabilitation is usually applied by an audiologist and sometimes also by an occupational physician, social worker, psychologist, or speech therapist. Based on the patients’ needs, the rehabilitation consists of interventions belonging to one or more of the four components mentioned above. Although it is increasingly acknowledged that it is important to address patient’s work needs in aural rehabilitation (Granberg and Gustafsson 2021; Zuriekat et al. 2021), due to the lack of literature, it is unclear to what extent these kinds of services are currently provided and what the effects are of current services. To the best of our knowledge, a prospective evaluation of the NFR after the provision of any kind of aural rehabilitation has only been conducted in two recent studies (Gussenhoven et al. 2017; van Leeuwen et al. 2021).

Gussenhoven et al. (2017) performed a randomized controlled trial comparing a multidisciplinary program of aural rehabilitation including vocational and audiological components with audiological care as usual. They included employees experiencing hearing difficulties and restrictions at work due to their hearing loss. No significant decrease in the NFR was found in both groups at 3, 6, 9, or 12 month follow-up, and the effect of the intervention on the NFR did not differ between the two groups. Van Leeuwen et al. (2021) performed a cohort study and evaluated the effect of using hearing aids and/or hearing assistive listening devices on the NFR. They included employees aged 18–67 with normal hearing or with hearing loss. A total of 147 employees with hearing loss were included who did not use hearing aids nor hearing assistive listening devices at baseline, but would be eligible for hearing aids based on their result on an online digit-triplet speech in noise test. After 5 years, 29 of them reported to use hearing aids and/or hearing assistive listening devices and 118 were not. Van Leeuwen et al. (2021) concluded that the uptake of hearing aids and/or hearing assistive devices did not have a significant effect on NFR.

It can thus be concluded that a positive effect of aural rehabilitation on the NFR has not yet been demonstrated. Also, there are no studies available investigating factors associated with change in the NFR of employees with hearing loss who receive aural rehabilitation. Such research would be useful for evaluating and optimizing the aural rehabilitation strategies that are currently used. Therefore, the study objectives are:

- To determine whether the model of van der Hoek-Snieders et al. (2020) can be confirmed in a different population regarding the factors influencing the NFR and LE in employees with hearing loss;
- To assess the NFR of employees with hearing loss before and after aural rehabilitation;
- To examine to what extent change in the NFR can be explained by changes in subjective listening effort, personal adjustments, communication strategies, auditory work demands, and self-reported hearing ability.

## Methods

### Study design

This prospective study was performed in employees with hearing loss who received aural rehabilitation at two audiological centers in the Netherlands, respectively at one location of the Amsterdam University Medical Center (UMC) and at three locations of Libra Revalidation and Audiology. Outcomes were measured by an extensive online questionnaire at baseline (*T*_0_) and 3 months follow-up (*T*_1_). Between *T*_0_ and *T*_1_, patients received different components of aural rehabilitation.

### Participants

**Fig. 2:**
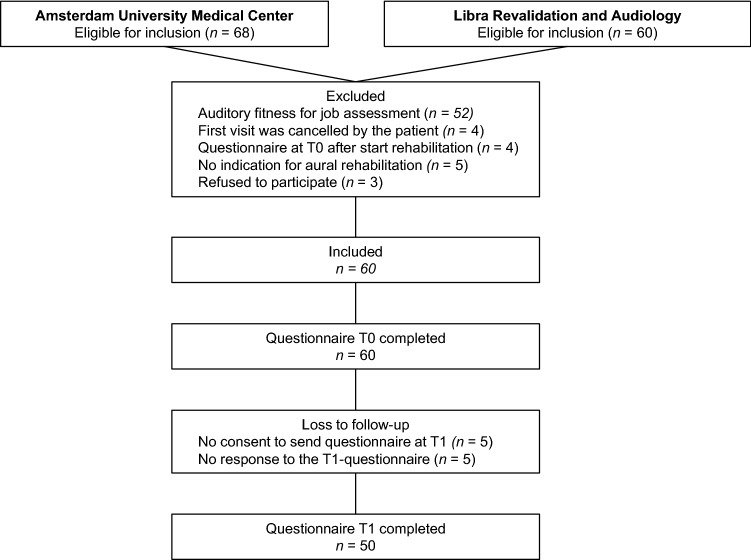
Flowchart showing the participants that were included in this study, exclusions, and the completion of the questionnaires at *T*_0_ and *T*_1_

Eligible patients were referred to the audiological center of the Amsterdam UMC between 2019 and 2021 or the audiological center of Libra Revalidation and Audiology between 2020 and 2022. The inclusion criteria further required patients to be aged between 18 and 67, to visit the audiological center because of hearing complaints in the work situation, and to provide informed consent for participating in this study. Hearing complaints in the working situation could either be the reason of the referral to the audiological center or these complaints were concluded after the intake with the audiologist. Eligible patients received information about the study and were asked consent for using their responses on the baseline questionnaire (part of the routine health care process) for this study, for sending a second survey for research purposes after 3 months, and for accessing their patient file to extract their pure-tone audiometry results and the type of intervention that was applied. Patients were excluded if the reason for their referral was an auditory fitness for job assessment, because these patients visit the audiological center to ensure that they can perform their job safely and effectively rather than to reduce their LE and NFR. Patients were also excluded if the first visit at the audiological center was cancelled, if the baseline questionnaire was not filled in or was filled in after the start of the intervention, and if there was no indication for aural rehabilitation (Fig. 2). The audiologist (routine clinical care) decided whether there was an indication for aural rehabilitation or not.

Table 1 shows the demographic and clinical characteristics of the patients.

**Table 1:** Baseline characteristics of the included participants (*N* = 60)

Variable	Mean (sd)	Range	No. (%)
Age in years	48.0 (11.3)	22–63	
Gender, male			18 (30.0)
General health condition, good			48 (80.0)
Educational level			
Lower vocational			1 (1.7)
General intermediate			4 (6.7)
Intermediate vocational			18 (30.0)
General secondary			2 (3.3)
Higher vocational			25 (41.6)
University			10 (16.7)
Work sector			
Healthcare and public welfare			20 (33.3)
Business and financial services			11 (18.3)
Education			12 (20.0)
Construction industry			7 (11.7)
Trade and catering			7 (11.7)
Other			3 (5.0)
Number of working hours	30.7 (8.1)	8–45	
Hearing aids, yes			37 (61.7)
Binaural hearing impairment	42.8 (19.9)	5.6–88.6	
Auditory work demands^a^	29.7 (7.0)	16–45	
SSQ^b^			
Speech	5.5 (1.7)	1.0–9.1	
Spatial	4.5 (2.1)	0.0–10.0	
Quality	6.6 (1.9)	1.75–10.0	
Need for recovery^c^	50.1 (21.8)	0–100	
Subjective listening effort^d^	10.0 (3.4)	2–18	
Feeling something should change, yes			21 (35.0)
Personal adjustments^e^	51.6 (12.9)	21–77	
Communication strategies^f^	68.5 (9.0)	45–86	
Self-acceptance mean item score	3.7 (1.0)	1.3–5.0	
Acceptance of loss mean item score	3.2 (0.9)	1.3–5.0	
Stress and withdrawal mean item score	3.0 (0.9)	1.1–4.9	

*SSQ* Speech, Spatial, and Qualities of hearing scale

^a^Higher score indicates higher auditory work demands

^b^Higher score on the SSQ indicates greater self-reported hearing ability

^c^Higher score indicates higher level of need for recovery

^d^Higher score indicates higher level of subjective listening effort

^e^Higher score indicates more adequate personal adjustments

^f^Higher score indicates more adequate communication strategies

### Aural rehabilitation

All patients received individually tailored aural rehabilitation.

Sensory management interventions could include the provision and fitting of hearing aids and other assistive listening devices, such as table microphones. We distinguished the provision and fitting of hearing aids in patients who did not use hearing aids at *T*_0_ (First HA), patients who used one hearing aid at *T*_0_ and received a second hearing aid (bilateral fitting), patients who used hearing aids at *T*_0_ and received new hearing aids (repeated fitting), and patients who used hearing aids at *T*_0_ of which the settings were optimized (fine-tuning HA). Sensory management interventions were provided by or under supervision of an audiologist.

Perceptual training could involve a speech reading training. This training could be provided individually or the patient could be referred for a group training. Perceptual training was provided by a speech therapist and a social worker.

Instruction and counselling were described as one category, because we expected that the subtle difference between instruction and counselling could not be recognized easily based on a patient file. The instruction/counselling could focus on coping—the development of effective listening strategies and coping behavior—or on work adjustments, such as adjusting working hours or environmental changes that improve room acoustics at the workplace. The instruction/counselling could be provided by an audiologist, psychologist, social worker, or an occupational physician.

We retrospectively derived the details of the provided aural rehabilitation components (sensory management, perceptual training, instruction/counselling) from patient files (Table 2). Regarding the component sensory management, we distinguished first HA, bilateral fitting, repeated fitting, fine-tuning HA, and listening devices. Regarding the component instruction/counselling, we distinguished whether there was a focus on coping or on work adjustments.

**Table 2:** Aural rehabilitation services that were provided in the study population (*N* = 60)

Hearing aid interventions	No other	Listening devises	Coping counselling	WA counselling	Listening devises & coping counselling	Listening devices & WA counselling	Perceptual training & coping counselling	Coping counselling & WA counselling
First HA (*n* = 14)	9	2	3	–	–	–	–	–
Bilateral fitting (*n* = 2)	–	1	1	–	–	–	–	–
Repeated fitting (*n* = 15)	4	6	2	–	–	2	–	1
Fine tuning HA (*n* = 23)	4	1	3	4	3	3	2	3
No HA intervention (*n* = 6)	1	–	2	–	1	–	–	2

*HA* Hearing aid, *WA* Work adjustments

### Questionnaires

At *T*_0_ and *T*_1_, patients received questionnaires by email. The questionnaires at *T*_0_ and *T*_1_ are the same, except for demographics that were only included at *T*_0_ (age, gender, general health condition, educational level, work sector, number of working hours). The questionnaires included questionnaires assessing the NFR, LE, “feeling that something should change”, personal adjustments, communication strategies, auditory work demands, and self-reported hearing ability.

The baseline questionnaires are routinely administered at the two audiological centers. The moment that patients receive these questionnaires slightly differ between the two centers. Patients who visited the Amsterdam UMC received the baseline questionnaire before the intake at the audiological center. Patients who visited Libra Revalidation and Audiology received the baseline questionnaire just after the intake with the audiologist. The follow-up questionnaires are not routinely administered at the audiological centers and were sent for research purposes.

All questionnaires were sent via the clinical management program Castor Electronic Data Capture (Castor EDC, Ciwit BV, Amsterdam, The Netherlands). This program complies with the Good Clinical Practice guidelines.

### Primary outcome measure

NFR was assessed using the NFR scale that is part of the Questionnaire on the Experience and Evaluation of Work 2.0 (QEEW 2.0) (van Veldhoven et al. 2015). This scale consists of six statements, such as “Because of my job, at the end of the working day I feel rather exhausted” and “I find it hard to show interest in other people when I have just come home from work”. These statements have four response categories, respectively: always, often, sometimes, or never. The sum score can be converted to a scale score that ranges from 0 to 100, with higher scores indicating higher levels of NFR.

### Secondary outcome measure

LE was inventoried with six questions on a 4-point response scale using the Amsterdam Checklist for Hearing and Work. The questions concern the effort it takes to perform six hearing-related job activities, respectively, detecting sounds, distinguishing sounds, communication in quiet, communication in noise, localizing sounds, and being exposed to loud sounds. In accordance with van der Hoek-Snieders et al. (2020), a sum score was calculated of these six questions. This score can vary between 0 and 18.

### Determinants

#### Feeling that something should change

“feeling that something should change” was assessed with a single, dichotomous question: do you feel that something should change in your work situation?

#### Personal adjustments and communication strategies

The shortened and validated version of the Communication Profile for the Hearing Impaired (CPHI) was used to assess personal adjustments and communication strategies (Mokkink et al. 2010). This questionnaire aims to distinguish between adequate and inadequate coping behavior in people with hearing loss. The domain personal adjustments consists of three scales, respectively, self-acceptance (4 questions), acceptance of loss (3 questions), and stress and withdrawal (9 questions). Questions include statements, such as “I feel ashamed if I have to ask someone to repeat himself” (self-acceptance), “I find it difficult to accept that I am hard of hearing (acceptance of loss), and “I often withdraw because of my hearing loss” (stress and withdrawal). The domain communication strategies consists of three scales, respectively, maladaptive behavior (7 questions), verbal strategies (7 questions), and non-verbal strategies (5 questions). Questions include statements, such as “I avoid conversations with strangers, because of my hearing loss” (maladaptive behavior), “I have asked my friends and colleagues to attract my attention before talking to me” (verbal strategies), and “I always try to watch a persons’ face” (non-verbal strategies). Responses are given on a 5-point scale with higher scores indicating more favorable coping strategies. Part of the statements has a frequency response scale (almost never, sometimes, regularly, usually, almost always) and the other statements have an agreement response scale (strongly disagree, disagree, uncertain, agree, strongly agree). We calculated the sum score of the personal adjustments and communication strategies scales according to van der Hoek-Snieders et al. (2020), and the mean item score of the six subscales.

#### Auditory work demands

Using the Amsterdam Checklist for Hearing and Work, the occurrence of six hearing-related job activities was inventoried on a 4-point response scale. A weighted sum score for auditory work demands was calculated according to van der Hoek-Snieders et al. (2020). Communication in quiet and distinguishing sounds received a weighting of 1, detecting sounds and localizing sounds received a weighting of 2, and being exposed to loud sounds and communication in noise received a weighting of 3. This score can vary between 0 and 48. The psychometric properties of this part of the Amsterdam Checklist for Hearing and Work have not been investigated yet.

#### General health condition

Patient’s general health condition was inventoried with a single question: how is your general health condition? Response categories were good, moderate, and poor. In accordance with van der Hoek-Snieders et al. (2020), the answers to this question were dichotomized for the statistical analysis (good versus moderate/poor).

#### Binaural hearing impairment

Pure-tone audiometry was performed as part of the routinely health care at the audiological centers. The degree of hearing loss was quantified by calculating the Binaural Hearing Impairment (BHI), defined as the mean pure-tone thresholds for air conduction at 1000, 2000, and 4000 Hz with a 5:1 weighting favoring the better ear (American Academy of Otolaryngology (Committee on Hearing 1979).

#### Self-reported hearing ability

Self-reported hearing ability was assessed with the Speech, Spatial, and Qualities of hearing scale (SSQ) (Gatehouse and Noble 2004). We used the Dutch version 3.2.1 (2007) that is also available in a shortened form: 17 questions, divided into three domains (Knoop et al. 2021). The first domain, speech comprehension (7 questions), assesses the ability to understand speech in different situations, such as situations in silence, with competing speakers, or in situations with continuous noise. In the second domain, spatial hearing (3 questions), the ability to locate sounds is measured as well as the ability to estimate the distance of sounds. The third domain, quality of hearing (7 questions), assesses the ease of listening, and the naturalness, clarity, and recognizability of different sounds. For each question, the self-rated ability is reflected by a score between 0 and 10, on a visual analogue scale, with higher scores reflecting greater ability (less disability). The average score was calculated for the three scales separately. Due to a programming error, the last question (‘Can you easily ignore other sounds when trying to listen to something?’) was not included in our questionnaire. Therefore, the average score of the quality of hearing scale and the average of all questions was calculated without considering the answer on this question.

### Statistical analysis

Patients’ baseline characteristics (Table 1) were described using descriptive statistics, as well as the components of aural rehabilitation that were provided (Table 3). We used histograms to check if the assumption of normality was fulfilled for the outcomes NFR, LE, personal adjustments, communication strategies, BHI, auditory work demands, and self-reported hearing ability.

**Table 3:** Correlations and prediction intervals comparing the correlations found in this study and the correlations found in the previous study by van der Hoek-Snieders et al. (2020)

	Previous study		Current study	
	Correlation	Prediction interval	Correlation	Hypothesis confirmed?
Need for recovery				
Feeling something should change	0.48	0.27; 0.68	0.46	Yes
Subjective listening effort	0.53	0.34; 0.72	0.54	Yes
General health condition	0.45	0.23; 0.66	0.33	Yes
Personal adjustments	−0.46	−0.71; −0.20	−0.37	Yes
Subjective listening effort				
Feeling something should change	0.39	0.16; 0.62	0.46	Yes
Binaural hearing impairment	0.32	0.07; 0.56	0.07	Yes
Auditory work demands	0.41	0.18; 0.63	0.58	Yes
Personal adjustments	−0.44	−0.69; −0.18	−0.56	Yes

To verify whether the patients of the Amsterdam UMC could be analyzed together with the patients of Libra Revalidation and Audiology, *t* tests were performed to evaluate group differences. No differences were found in the demographic and clinical characteristics between the patients who visited the Amsterdam UMC and the patients who visited Libra Revalidation and Audiology. Therefore, the results of all patients were described and analyzed together.

To assess the first research question, correlation coefficients were calculated between NFR/LE and the factors of the model. We calculated the correlation coefficients in the same way as in our previous study (van der Hoek-Snieders et al. 2020). The Pearson correlation coefficients were used to calculate the correlation between two continuous variables and bi-serial correlation coefficients (Kraemer 2014) were used to calculate the correlation between a continuous and a dichotomous variable. The interpretation of the correlation coefficients was weak (<0.3 or >−0.3), moderate (between 0.3 and 0.7 or between −0.3 and −0.7), or strong (>0.7 or <−0.7) (Ratner 2009). According to Spence and Stanley (2016)**,** we calculated 95% prediction intervals around the correlations found in the previous study. This calculation was based on a replication sample size of 60, which corresponds to the sample size of our study.

To achieve the second research objective, the smallest detectable change in the NFR was calculated for our study sample size according to Hoofs et al. (2017), and it was evaluated whether the effect size exceeded this value. Also, the differences between scores over time were calculated for the variables NFR, LE, personal adjustments, communication strategies, auditory work demands, and self-reported hearing ability. Paired *t* tests were used to evaluate differences between *T*_0_ and *T*_1_. Change scores were not calculated for the variables general health condition and the BHI, because the aural rehabilitation was not expected to change these variables. Change scores were also not calculated for the variable “feeling that something should change”, since differences in this variable would be difficult to interpret at group level. For example, increased need for change might reflect an unsatisfactory result of the rehabilitation, but might also reflect increased awareness of the impact of work circumstances on the hearing loss complaints. In a secondary analysis, the differences between the subscales from the CPHI were calculated and assessed using paired *t* tests.

We performed a post hoc power analysis based on the effect size of NFR. Our sample size would give a power of 74% and 5% significance in a paired mean comparison test.

To identify the factors associated with a decrease in NFR and LE, regression analyses were performed using the change scores (outcome and determinants). Every determinant was used separately in a univariate regression model and hierarchical multiple analyses were performed. For the primary and the secondary outcome measures, the first block consisted of the potential confounders age, gender, educational level, and BHI. In the next blocks, the determinants were added one by one. For each block, we calculated the change in amount of variance.

Data were analyzed using the Statistical Package for Social Sciences (SPSS) version 26.0 (Armonk New York USA). For all tests, the type I error was set to 0.05 and all tests were two-sided.

## Results

Table 2 presents comparisons between the correlation coefficients presented by van der Hoek-Snieders et al. (2020) and the correlation coefficients that were found in the current study. In line with the previous findings, NFR was moderately associated with “feeling that something should change” (*r* = 0.46, *p* < 0.01), LE (*r* = 0.54*, p* < 0.01), general health condition (*r* = 0.33, *p* = 0.01), and personal adjustments (*r* = −0.37*, p* < 0.01).

In accordance with the previous findings, LE was moderately associated with “feeling that something should change” (*r* = 0.46,* p* < 0.01), auditory work demands (*r* = 0.58, *p* < 0.01), and personal adjustments (*r* = −0.56, *p* < 0.01). A non-significant association was found between LE and the BHI (*r* = 0.07, *p* = 0.63).

Table 4 shows the mean scores and standard deviations of the questionnaire scores before and after the aural rehabilitation. Based on a sample size of 60, the smallest detectable change in NFR is 5.77. NFR decreased on average by 6.86.

**Table 4:** Paired *t* tests of questionnaire scores before and after receiving aural rehabilitation (*n* = 50)

	Baseline (*T*_0_)	Follow-up (*T*_1_)	Difference (*T*_1_−*T*_0_)	*t*	*P*
Need for recovery^a^	51.89 (20.47)	45.03 (21.62)	−6.86 (14.09)	−3.34	<0 .01
Subjective listening effort^b^	9.90 (3.33)	8.30 (3.72)	−1.60 (3.03)	−3.73	<0.01
Personal adjustments^c^	51.59 (12.87)	56.37 (13.66)	4.78 (7.68)	3.27	<0.01
Communication strategies^d^	68.17 (9.23)	69.36 (10.00)	1.19 (7.77)	0.99	0.33
Auditory work demands^e^	29.38 (6.81)	30.10 (6.81)	0.72 (4.31)	1.18	0.24
SSQ speech^f^	5.51 (1.73)	6.16 (1.78)	0.65 (1.39)	2.76	<0 .01
SSQ spatial^f^	4.46 (2.19)	5.16 (2.28)	0.70 (1.66)	2.56	0.02
SSQ quality^f^	6.57 (1.86)	6.91 (1.68)	0.34 (1.40)	1.46	0.15

*SSQ* Speech, Spatial, and Qualities of hearing scale

^a^Higher score indicates higher level of need for recovery

^b^Higher score indicates higher level of subjective listening effort

^c^Higher score indicates more adequate personal adjustments

^d^Higher score indicates more adequate communication strategies

^e^Higher score indicates higher auditory work demands

^f^Higher score indicates greater self-reported hearing ability

A significant difference was found for the variables NFR, LE, personal adjustments, SSQ speech, and SSQ spatial. No significant differences were found regarding the variables communication strategies, auditory work demands, and SSQ quality.

The secondary analysis revealed a significant difference for two of the three personal adjustment subscales, respectively, for acceptance of loss and for the subscale stress and withdrawal (Table 5). No significant differences were found in the three communication strategies subscales.

**Table 5:** Paired *t* tests of CPHI subscales before and after receiving aural rehabilitation (*n* = 50)

	Baseline (*T*_0_)	Follow-up (*T*_1_)	Difference (*T*_1_−*T*_0_)	*t*	*p*
Personal adjustments					
Self-acceptance^a^	3.70 (1.04)	3.91 (0.91)	0.21 (0.72)	1.87	0.07
Acceptance of loss^a^	3.17 (0.93)	3.62 (1.67)	0.45 (1.32)	2.87	0.01
Stress and withdrawal^a^	3.05 (0.88)	3.28 (0.98)	0.23 (0.51)	2.17	0.04
Communication strategies					
Maldaptive behavior^a^	4.02 (0.76)	4.07 (0.73)	0.05 (0.53)	0.54	0.59
Verbal strategies^a^	2.97 (0.89)	3.09 (0.89)	0.12 (0.76)	1.01	0.32
Non-verbal strategies^a^	3.84 (0.96)	3.84 (0.85)	0.00 (0.64)	0.10	0.92

*SSQ* Speech, Spatial, and Qualities of hearing scale

^a^Higher scores indicate more adequate coping behavior

**Fig. 3:**
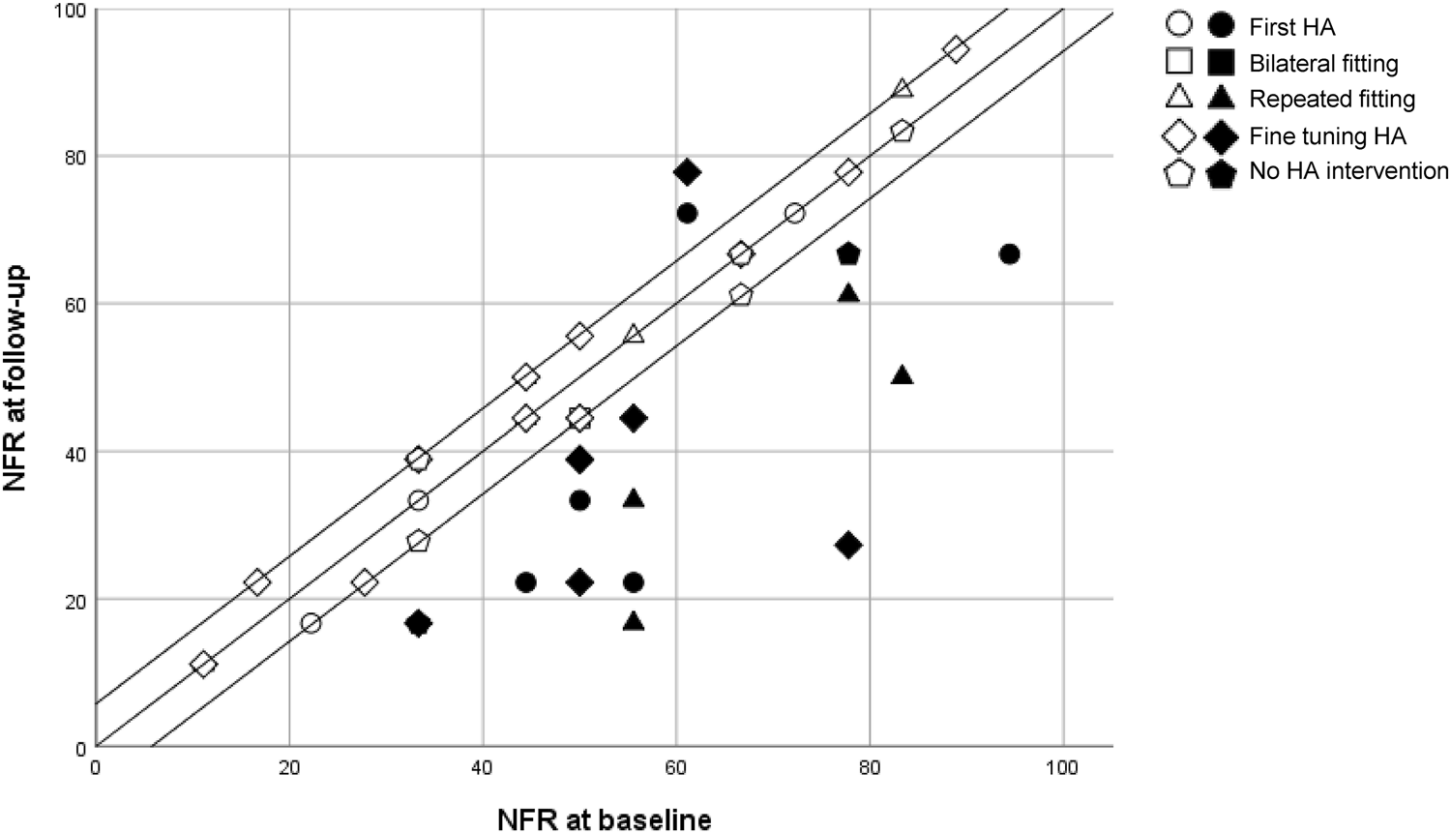
Scatterplot of Need For Recovery (NFR) scores at *T*_0_ and *T*_1_ for employees who received different hearing aid interventions. Icons on the diagonal represent NFR scores that were exactly the same at *T*_0_ and *T*_1_. The two other lines show the smallest detectable change of 5.77. The icons are colored in for the employees in which the NFR score had changed more than the smallest detectable change.

In 29 patients, the difference between the NFR score at *T*_0_ and *T*_1_ was 5.77 or less (Fig. 3). In 2 patients, the NFR scores increased more than 5.77. In 16 patients, the NFR score decreased more than 5.77. There were no obvious differences in the improvement in NFR between patients receiving different hearing aid interventions.

In the univariate regression analyses (Table 6), three change scores were found to significantly explain variance in the NFR change score, respectively, LE (*p* = 0.03), personal adjustments (*p* < 0.01), and SSQ quality (*p* < 0.01). In the hierarchical regression analyses, the amount of variance changed significantly when the determinants subjective listening effort, personal adjustments, and SSQ quality were added to the model (Table 7). The amount of explained variance was highest when the personal adjustments were added to the model. This model explained 53% of the variance in the change in NFR.

**Table 6:** Results of the univariate regression analysis of the factors associated with change in the Need For Recovery (NFR) and subjective listening effort (LE)

Outcome	Determinant	*B*	95% CI	*p*	*R*^2^
Change in need for recovery	Subjective listening effort	1.40	−9.12; −0.19	0.03	0.10
	Personal adjustments	−1.23	−1.69; −0.78	<0.01	0.45
	Communication strategies	0.02	−0.56; 0.60	0.94	0.00
	Auditory work demands	−0.01	−0.97; 0.99	0.98	0.00
	SSQ speech	−2.12	−5.21; 0.97	0.17	0.04
	SSQ spatial	−1.95	−4.39; 0.85	0.14	0.06
	SSQ quality	−5.24	−7.82; −2.66	<0.01	0.28
Change in subjective listening effort	Personal adjustments	−0.13	−0.56; −0.01	0.04	0.11
	Communication strategies	−0.11	−0.26; 0.02	0.09	0.07
	Auditory work demands	0.25	0.06; 0.44	0.01	0.12
	SSQ speech	−1.08	−1.70; −0.47	<0.01	0.22
	SSQ spatial	−0.59	−1.12; −0.08	0.03	0.10
	SSQ quality	−0.71	−1.34; −0.09	0.03	0.11

*SSQ* Speech, Spatial, and Qualities of hearing scale

**Table 7:** Results of the hierarchical multiple regression analysis of the factors associated with change in Need For Recovery (NFR) and subjective listening effort (LE)

Outcome	Block	Predictors	*R*^2^	*R*^2^ change**	*p* *R*^2^ change
Change in need for recovery	1	Age Gender Educational level BHI	0.03	–	–
	2*	Subjective listening effort	0.23	0.20	<0.01
	3*	Personal adjustments	0.53	0.50	<0.01
	4*	Communication strategies	0.04	0.01	0.55
	5*	Auditory work demands	0.03	0.00	0.75
	6*	SSQ speech	0.15	0.12	0.05
	7*	SSQ spatial	0.13	0.10	0.10
	8*	SSQ quality	0.38	0.30	<0.01
Change in subjective listening effort	1	Age Gender Educational level BHI	0.06	–	–
	2*	Personal adjustments	0.15	0.10	0.05
	3*	Communication strategies	0.08	0.02	0.31
	4*	Auditory work demands	0.12	0.06	0.09
	5*	SSQ speech	0.26	0.20	<0.01
	6*	SSQ spatial	0.13	0.07	0.09
	7*	SSQ quality	0.15	0.09	0.05

*SSQ* Speech, Spatial, and Qualities of hearing scale

^*^The determinants of block 1 were included as potential confounders in the other blocks

^**^R^2^ change in comparison to block 1

All change scores, except for the communication strategies change score, were found to significantly explain variance in the LE change score in the univariate regression analyses, respectively, personal adjustments (*p* = 0.04), auditory work demands (*p* = 0.01), SSQ speech (*p* < 0.01), SSQ spatial (*p* = 0.03), and SSQ quality (*p* = 0.03). In the hierarchical regression analyses, the amount of variance changed significantly when the SSQ speech was added to the model (Table 7). This model explained 12% of the variance in the change in LE.

## Discussion

The aim of this study was twofold, respectively, to determine whether the model of van der Hoek-Snieders et al. (2020) could be confirmed regarding the factors influencing the NFR and LE in employees with hearing loss and to identify the factors associated with a decrease in NFR and LE after 3 months of aural rehabilitation.

Analysis of the baseline data confirmed the relationships in the model of factors influencing the NFR, since all correlation coefficients were consistent with the previous study. Our results therefore support the conceptual premise that higher LE can be an explanation of increased NFR after work (Kramer et al. 2006). However, in agreement with the model, our results suggest that this explanation is not conclusive, and that increased NFR can also partially be explained by the way employees cope with their hearing loss. The hypotheses regarding the outcome LE were also confirmed, but it must be noted that the association with BHI was weak and non-significant. Although employees with hearing loss have been shown to report higher LE compared to those with normal hearing (Kramer et al. 2006), our results do not indicate that differences in the degree of hearing loss can explain the severity of the LE. An explanation is that the degree of hearing loss was moderate in the majority of the study participants. The differences in degree of hearing loss were thus relatively small. Also, the degree of limitations does not only depend on the degree of hearing loss, but also on other factors, such as the auditory work demands or the personal adjustments (van der Hoek-Snieders et al. 2020). Finally, the association between the degree of hearing loss and LE would possibly be higher when the degree of hearing loss is measured with a performance test in an aided listening situation. This should be assessed by future research.

Analysis of the questionnaire data before and after the aural rehabilitation revealed significant improvements, both in NFR and in LE. Our study is the first that demonstrates that the NFR of employees with hearing loss can be improved by aural rehabilitation. In previous studies, no significant improvement in NFR was reported after aural rehabilitation (Gussenhoven et al. 2017; van Leeuwen et al. 2021). An explanation might be that the population of Gussenhoven et al. (2017) included a relatively high number of participants with low NFR, which might have resulted in a floor effect in their study. The mean NFR hardly differs between our study (mean = 50.1, SD = 21.6) and the study of Gussenhoven et al. (2017) (mean = 46, SD = 31). However, employees presented substantially more often with low NFR (NFR score below 20) in the latter study. Specifically, low NFR was found in 8% of participants in our study and in 26% of the participants in the study of Gussenhoven et al. (2017). The number of employees with low NFR is not mentioned by van Leeuwen et al. (2021). Differences in follow-up time might also explain the finding that we found a significant reduction in NFR in contrast to earlier studies. Our follow-up time was 3 months, whereas van Leeuwen et al. (2021) had a follow-up time of 5 years. It could therefore be the case that NFR decreases directly after the aural rehabilitation, but increases again after some time. A similar pattern was observed in a recent study, including patients that received their first hearing aid (Holman et al. 2021b). Although listening related fatigue decreased from before fitting to 6 months post-fitting for some of the included patients, no change was observed in long-term general fatigue. This pattern was however not concluded by Gussenhoven et al. (2017) who had a follow-up time of 3, 6, 9, and 12 months, and should be investigated by future research. Differences in the provided intervention between the studies might also explain that we found a significant reduction in NFR in contrast to earlier studies, such as differences in the aural rehabilitation decisions, the type of counselling, and the quality of the technology that was used. Although the aspects of aural rehabilitation that were provided differed between the patients in this study, most patients in our study received a broad intervention including several aspects of aural rehabilitation. For example, instruction or counselling on coping behavior was provided to 31% of our study population, to 14% of the intervention group of Gussenhoven et al. (2017), and van Leeuwen et al. (2021) did not assess this aspect of aural rehabilitation. Presumably, instruction or counselling on coping behavior was provided more frequently in our study than in the two previous studies.

Although the mean NFR decreased after the aural rehabilitation, NFR only decreased in approximately one-third of the employees. This finding suggests that the current usual practice may not be sufficient to achieve a reduction in NFR in all employees with hearing loss. Therefore, improving current practices should be considered and investigated. Also, there is need for standards or guidelines of hearing health care for employees with hearing loss. For example, the use of questionnaires regarding NFR, LE, and hearing-related coping behavior at baseline seems to be useful and convenient to describe patient’s work needs at baseline. However, these questionnaires need to be validated for the use of diagnosing and evaluating the hearing-related difficulties of employees with hearing loss. Also, in our study sample, hearing aid interventions received most attention, whereas the application of assistive listening devices and the use of instruction/counselling was not that often registered. Although this is in line with international practices (Hickson et al. 2013; Kochkin 2009; Timmer et al. 2015), the great focus on hearing aid interventions might not have resulted in the optimal mix of aural rehabilitation components.

We did not observe obvious differences in the improvement in NFR between patients receiving different hearing aid interventions. Although it would be plausible that the provision of a first hearing aid would have greater impact on NFR than fine-tuning hearing aid settings, this appears not to be the case in our study population. This might imply that the effect of hearing aid interventions on NFR might be rather marginal, which is in line with results of van Leeuwen et al. (2021). Since the follow-up time of 3 months was relatively short, it might also be the case that the first hearing aid users were not yet used to their hearing aid, which might have suppressed its effect on the NFR. Another explanation is that hearing aids may not always meet the expectations of first hearing aid users. In that case, managing patients expectations on what effects can realistically be expected from hearing aids might improve rehabilitation outcomes. Future studies with greater sample size and longer follow-up time should further assess this matter.

Our regression analysis revealed that change in NFR and LE can best be explained by different factors. Change in NFR could best be explained by change in personal adjustments, whereas change in LE could best be explained by change in self-reported hearing ability. This finding suggests that improved hearing might result in decreased LE, but not automatically in decreased NFR. Especially, interventions that affect personal adjustments may be promising to reduce NFR in employees with hearing loss. As suggested in previous studies (Backenroth-Ohsako et al. 2003; Gussenhoven et al. 2017; van Leeuwen et al. 2021), we therefore hypothesize that greater improvement in NFR might be obtained when sensory management interventions are not provided in isolation, but combined with interventions that foster adequate coping behavior. Future research is required to assess this hypothesis, since no conclusions on causality can be drawn because of the design of this study.

Some strengths and limitations should be noted for this study. Due to a programming error, one SSQ question was not included in the questionnaire. We do not expect that this has had a major impact on the SSQ spatial score, because this scale score is an average score of 7 questions. Also, since the last question was missing, this cannot have influenced the scores of other questions.

This study was performed in the setting of routine clinical practice, which improves the applicability of the results. A downside of our design was that no homogeneous intervention was provided and that there was no control group. Therefore, we cannot conclude that the improvement in NFR can be attributed to (aspects of) the aural rehabilitation. Also, the study population was too small to run subgroup analyses on patients who received the same intervention. The post hoc power analysis that was based on the effect size of NFR revealed that the 80% power was not achieved. This implies that our study might have been slightly underpowered to detect changes in the NFR. Despite this, we found a significant difference in the NFR.

We carefully described the components of aural rehabilitation that were provided using patient files, but we may have missed some aspects of the provided rehabilitation. For example, audiologists of the included audiological centers often give some kind of instruction on how the hearing aids or assistive listening devices function and how they can be properly used. However, this type of instruction was not administered in the patient files, and is therefore not reported in this study. Another study limitation is that the follow-up time of this study was relatively short. Aural rehabilitation was provided within this period in most, but not in all patients, which resulted in the exclusion of a few patients. An advantage of the follow-up time of 3 months is that there is a smaller chance that the NFR has changed due to other reasons than the aural rehabilitation.

## Concluding remarks

The NFR and LE of employees with hearing loss can be improved by aural rehabilitation, but this study shows that this is true in only part of the employees. Therefore, improving current practices should be considered and evaluated, for example by applying a different combination of rehabilitation components. Especially, interventions that affect personal adjustments may be promising to further reduce the NFR in employees with hearing loss.
